# A Case of Incomplete Removal of Horseshoe Kidney by Laparoscopic Nephrectomy in an Adult Leading to Urinary Leak: An Eye Opener

**DOI:** 10.1155/2019/4132521

**Published:** 2019-05-02

**Authors:** S. Venkat Ramanan, P. Velmurugan, A. R. Bhaskar Prakash, Anuj Arora, LeelaKrishna Karri

**Affiliations:** Department of Urology, Sri Ramachandra Medical College and Research Institute, Chennai, India

## Abstract

Horseshoe kidney is a common congenital fusion anomaly of the kidneys. It poses a challenge to surgeon because of its very variable anatomy in terms of location and lie, fusion, pelvicalyceal system, and the renal vessels. Here we describe a case of laparoscopic nephrectomy in a horseshoe kidney complicated by incomplete removal because of not realizing the lower and medial extent of pelvicalyceal system across midline to the contralateral side leading to persistent urine leak. The relevant anatomy and the necessary precautions to prevent such complication have been discussed.

## 1. Case Report

**Figure 1:**
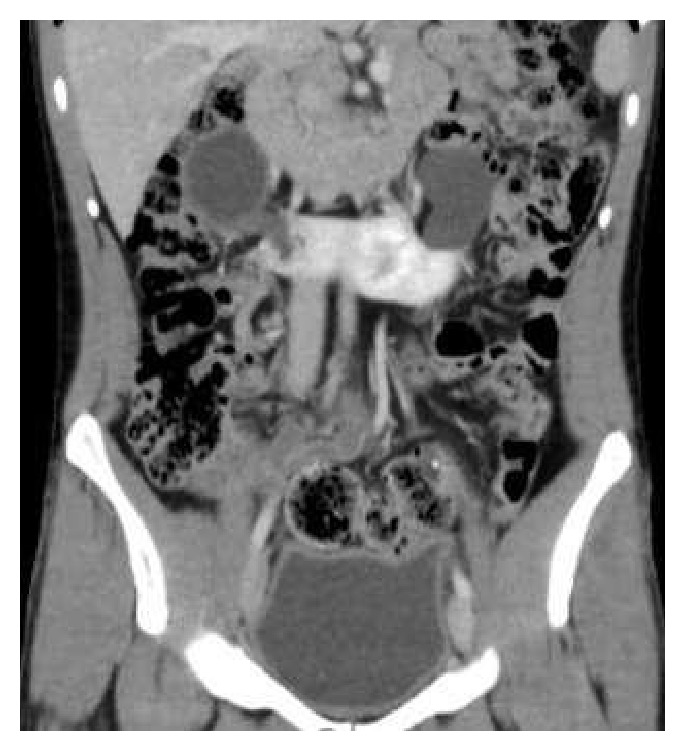
Initial preoperative CE-CT scan (coronal section) showing horseshoe kidney with right hydronephrosis.

**Figure 2:**
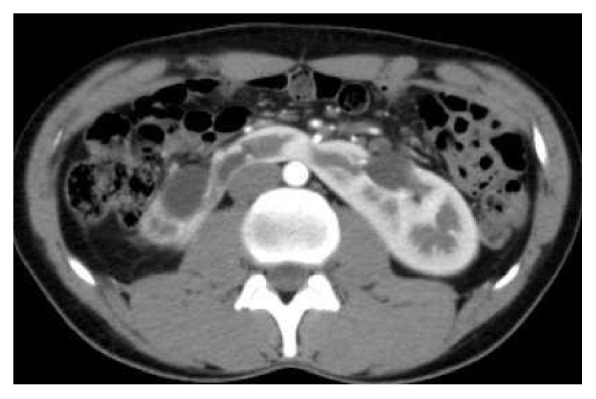
Initial preoperative CE-CT scan (axial section) showing horseshoe kidney with the fusion of the lower poles to the left of aorta.

**Figure 3:**
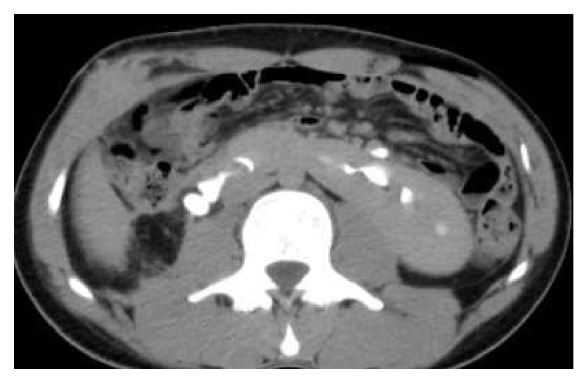
CE-CT following laparoscopic right nephrectomy showing leakage of contrast from the residual stump of the right lower pole.

**Figure 4:**
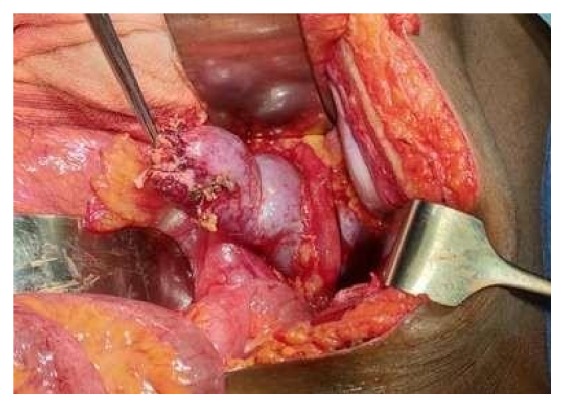
Intraoperative picture of exploratory laparotomy showing small residual stump of lower pole of right kidney fused to the lower pole of left kidney. Left ureter is seen running over the lower pole of left kidney.

A 25-year-old man, driver by profession, presented with recurrent episodes of dull aching right flank pain of 6-year duration. The pain was affecting his work. The patient was thin built with a body-mass-index of 20 Kg/m^2^. The preoperative CT scan revealed a horseshoe kidney (HSK) with the right side involved by ureteropelvic-junction obstruction (UPJO) causing gross hydronephrosis and parenchymal thinning (Figures 1 and 2). The relative renal function was 11% on DTPA renogram. The left renal moiety was functioning well with a GFR of 71.9 ml/min. The S. Creatinine was 0.9 mg%. The patient also had a history of epilepsy but there was no identifiable congenital anomaly other than the HSK. The options of right pyeloplasty and right nephrectomy with their pros and cons were discussed with the patient. The patient opted for nephrectomy. A transperitoneal laparoscopic right nephrectomy was done using five ports in the right lateral position. A preliminary retrograde pyelography (RGP) or right ureteric catheterization was not done. The right colon and duodenum were reflected medially to expose the kidney. As expected the right kidney was lying relatively lower and medially than what a normal kidney would be. Other than the main renal artery, the upper and the lower poles were supplied by accessory polar artery each. The upper polar artery itself had a very early branching and the lower polar artery was crossing the UPJ and causing obstruction. There were two right renal veins lying between the main renal artery and the lower polar artery. The right ureter was divided about 4 cm below the ureteropelvic junction. All the arteries and the veins were clipped with hem-o-lok clips and divided. The kidney was dissected within the Gerota's fascia from the upper pole downwards. The isthmus was mobilized to the extent possible and was divided just to left of inferior vena cava. Body side of the cut edge showed some brisk bleeding. The cut edge was sutured with 2'0 V-Loc sutures over a surgical bolster and hemostasis was secured. The specimen was removed using an endo-bag and a drain was placed. There was no intraoperative complication. The only abnormal event in the postoperative period was a persistent drain output of 100ml of urine every 24 hours. The drain fluid creatinine was 22mg%. The postoperative ultrasound examination of the abdomen did not reveal any significant intra-abdominal collection. Left retrograde pyelogram and right retrograde ureterogram done on the 14th postoperative day (POD) did not reveal any contrast leak either from the left side or from the right ureteric stump. Subsequently CT urogram was done which showed that a small residual stump of the right kidney fused to the lower pole of the left kidney was still viable and producing and leaking urine (Figure 3). This stump was supplied by a tiny arterial twig from the left renal artery. An exploratory laparotomy was done on the 16th POD through upper midline incision. The descending colon was reflected medially to expose the lower pole of the left kidney with the attached stump of the right kidney. The stump was densely adherent to the IVC and surrounding structures. For additional exposure and dissection, the mesentery of the transverse colon was opened lateral to the inferior mesenteric vein. The stump was freed from the IVC and delivered to the left side from underneath the inferior mesenteric artery (Figure 4). The stump was divided flush with the lower pole of the left kidney and the cut edge was sutured with 2'0 Vicryl over surgical bolster. The postoperative recovery was uneventful.

## 2. Discussion

**Figure 5:**
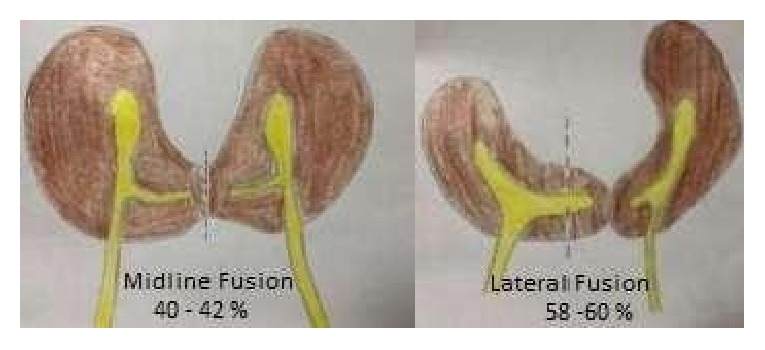
Midline lower polar fusion (40 to 42%) and lateral lower polar fusion (58% to 60%).

Horseshoe kidney is the commonest fusion anomaly of the kidneys and accounts for more than 90% of it [1, 2]. The reported incidence based on autopsy and radiographic data is 1:350 to 1:666 with a 2:1 male predominance [3–7]. HSK differs from crossed fused renal ectopia in that, unlike the latter in which only one kidney moves abnormally across the midline to fuse with the contralateral kidney, in HSK, both of the kidneys migrate abnormally and fuse. In 95% of cases the kidneys are fused at the lower poles. The lower polar fusion occurs in midline in 40 to 42% of cases giving rise to U-Shaped HSK or Symmetric HSK (Figure 5). In the remaining 58% to 60% of cases the fusion is lateral to the midline with one kidney being more horizontal and extending across the midline to fuse with the lower pole of the more vertical contralateral kidney (asymmetric HSK or L-shaped HSK) [7, 8]. The isthmus is composed of functioning renal parenchyma in majority of cases with only 4.4% to 15% of HSKs showing a fibrous isthmus [7, 9]. In 70% of the cases there are multiple renal vessels with variable origin and drainage patterns. The challenges in laparoscopic nephrectomy of a HSK pertain to dealing with multiple vessels as well as disconnecting the kidney at the isthmus. The issue of multiple vessels and variable vascular patterns is well recognized and it calls for a meticulous delineation of the vascular anatomy both preoperatively and intraoperatively and taking control of these vessels appropriately during surgery [10, 11]. However, the same kind of awareness regarding the anatomy of the isthmus during isthmectomy seems to be lacking. The reports of isthmectomy in the literature do not specify the type of isthmus that has been dealt with [12, 13]. As pointed out earlier, about 60% of HSKs with lower polar fusion have L-shaped kidney with fusion happening well beyond the midline on the contralateral side. Reaching the point of fusion well beyond the midline during laparoscopy may not be easy and it may result in incomplete removal. If only a small stump of a parenchymatous isthmus is left behind it may not be of any consequence. However, if that parenchymatous stump was also to contain a small portion collecting system within, then urinary leak is a potential possibility. In our case the HSK was L-shaped with the lower pole of the right kidney reaching well beyond the midline to fuse with the lower pole of the left kidney lateral to the aorta. The point of fusion was just a linear line. The kidney was grossly hydronephrotic with the dilated calices eroding the parenchyma and reaching up to the edge of the kidney and fairly close to the point of fusion. But this anatomy was somehow not appreciated in the first CT-Urogram prior to laparoscopic nephrectomy. Even though the isthmus was divided medial to the IVC, it still left behind a small stump of parenchyma with its contained collecting system. This led to a persistent urinary leak. This adverse outcome in our case could have been averted by clearly identifying the type of fusion and the anatomy of the isthmus in the preoperative imaging. In case of any uncertainty in the preoperative imaging, retrograde pyelography prior to nephrectomy would have clarified the relationship of pelvicalyceal system and the isthmus and how far medially the PCS was extending. This knowledge would have helped in avoiding cutting through the collecting system when dividing the isthmus. Moreover, if the point of division of the isthmus has to be beyond the lateral side of the contralateral great vessel, then laparoscopic approach may be extremely difficult and an open surgery may be a better option to prevent any incomplete removal.
